# Hypereosinophilic syndrome with elevated IgG4 and T-cell clonality: A report of two cases

**DOI:** 10.1515/biol-2025-1138

**Published:** 2025-07-24

**Authors:** Meichun Huang, Limin Wang, Xiuxiu Li, Ying Lu

**Affiliations:** Renal Department, Tongde Hospital of Zhejiang Province, No. 234 Gucui Road, Hangzhou, 310012, China; Renal Department, Tongxiang First People’s Hospital, Tongxiang, 314500, China

**Keywords:** lymphocyte variant hypereosinophilic syndrome, IgG4, IgG4-related diseases, hypereosinophilia, acute kidney injury

## Abstract

In some cases, higher IgG4 levels are accompanied by increased circulating IgE, higher eosinophil counts, and various autoantibodies. Among these cases, IgG4-related disease (IgG4-RD) is one of the most frequently diagnosed conditions. This study reported two particularly complex and rare cases of hypereosinophilic syndrome (HES) associated with elevated IgG4 and T-cell clonality. The first case involved T-cell clonality complicated by HES and IgG4-RD, presenting with clinical features resembling eosinophilic granulomatosis with polyangiitis (EGPA). Laboratory findings showed a serum IgG4 concentration of 8.74 g/L, an IgG4/IgG ratio of 40.58%, and positive results for P-anti-neutrophil cytoplasmic antibodies (ANCA) and myeloperoxidase-ANCA. Renal biopsy findings were consistent with IgG4-related interstitial nephritis. However, routine hematological testing revealed a markedly elevated eosinophil count of 14.75 × 10^9^/L and eosinophilic infiltration in both lymph nodes and kidney tissue. Furthermore, monoclonal rearrangements of the T-cell receptor gamma and delta genes were identified. The second patient was ultimately diagnosed with HES with elevated IgG4 and T-cell clonality, with an elevated IgG4 concentration of 2.458 g/L and an eosinophil count of 14.75 × 10^9^/L. In conclusion, in cases presenting with elevated IgG4 levels and hypereosinophilia, further pathological and genetic evaluations may be essential to guide appropriate and timely treatment, improving patient prognosis.

## 1 Introduction

The IgG4 is the least abundant subclass of IgG in humans, with average circulating levels of only 0.003–0.004 g/L in healthy individuals. However, its levels frequently increase in the setting of tumors, infections, and immune-mediated diseases [1,2]. In some cases, elevated IgG4 levels are associated with increased circulating IgE, elevated eosinophil counts, various autoantibodies, and damage to the kidneys and other organ systems. However, the underlying causes of these changes often remain unclear [3].

The IgG4-related disease (IgG4-RD) is among the most frequently diagnosed conditions in such cases; however, its etiology is complex, and the pathological role of circulating IgG4 remains poorly understood. As a result, efforts to improve the diagnosis and management of these conditions are ongoing. The updated diagnostic criteria introduced in 2019 identify elevated eosinophil counts as an exclusion criterion [3,4]. According to the diagnostic criteria established in 2012, approximately 20–40% of patients meet these standards but often also present with elevated eosinophil counts. Eosinophilic infiltration of affected organs is observed in 51–86% of IgG4-RD cases. Elevated eosinophils and IgG4-RD are commonly accompanied by increased circulating IgE levels and allergic manifestations. In some rare instances, severe asthma has been the initial clinical presentation of IgG4-RD [5]. However, eosinophilia may result from the immune response intrinsic to IgG4-RD rather than concurrent allergic diseases. Importantly, elevated eosinophil counts and higher serum IgG4 concentrations have been closely linked to an increased risk of disease relapse [6].

Several other hematological disorders can also lead to elevated circulating eosinophil counts and serum IgG4 levels, contributing to multi-system involvement, particularly in conditions such as Kimura’s disease and lymphoproliferative disorders. However, reports of hematological clonal diseases associated with clonal hypereosinophilic syndrome (HES) and IgG4-RD are scarce [7,8]. Lymphocyte-variant HES (L-HES) is a rare clonal hematologic disorder primarily described in isolated case reports. Studies investigating the association between L-HES and IgG4-RD remain very limited.

The L-HES remains poorly understood, with no standardized diagnostic criteria currently established. It is characterized by clonal hypereosinophilia, leading to eosinophilic infiltration of various organs, abnormal lymphocyte populations, and clonal rearrangements of T-cell receptor (TCR) genes [9]. The diagnostic criteria for definite IgG4-RD include:

- Clinical evidence of diffuse or localized swelling or mass formation in one or multiple organs.
- Hematological findings show higher serum IgG4 concentrations (≥135 mg/dL).
- Histopathological features demonstrating: (1) dense lymphocyte and plasma cell infiltration with fibrosis, and (2) infiltration by IgG4-positive plasma cells, with an IgG4+/IgG+ cell ratio exceeding 40% and more than 10 IgG4+ plasma cells per high-power field (HPF).

Differentiating IgG4-RD from L-HES can be challenging. In a comparative study involving 31 patients with IgG4-RD and 13 patients with L-HES, peripheral blood eosinophilia was present in 8 of 31 IgG4-RD patients compared to all 13 L-HES patients. Elevated serum IgG4 levels were observed in 27 of 30 IgG4-RD patients but only in 5 of 12 L-HES patients. Serum IgE elevation was noted in 12 of 20 IgG4-RD patients and 8 of 13 L-HES patients. Flow cytometry revealed aberrant T-cell phenotypes in 7 of 23 IgG4-RD cases and all 13 L-HES cases. T-cell clonality detected by polymerase chain reaction was positive in 12 of 23 IgG4-RD patients and 10 of 13 L-HES patients. Both conditions may present with hypereosinophilia, elevated serum IgE and IgG4 levels, aberrant T-cell immunophenotypes, and T-cell clonality [10]. However, IgG4-RD is often associated with mass lesions and typically involves five or more organs, whereas L-HES usually affects no more than three organs.

The pathological mechanisms linking these two rare diseases remain unclear. In L-HES, abnormal lymphocyte gene rearrangements lead to clonal hypereosinophilia. Pathologically, L-HES is primarily characterized by eosinophilic infiltration, particularly involving the bone marrow, skin, respiratory tract, lymph nodes, and gastrointestinal system. Infiltration by lymphocytes and plasma cells is also commonly observed, which may contribute to elevated blood IgG4 levels. Eosinophilia in IgG4-RD is non-clonal and typically does not primarily affect lymph nodes or the skin. The hallmark pathological features of IgG4-RD include storiform fibrosis and an increased number of IgG4-positive plasma cells. Although eosinophilic infiltration is frequently present in IgG4-RD, it generally does not lead to prominent clinical symptoms [8,11].

However, reports of L-HES occurring alongside IgG4-RD are exceedingly rare. One case has been described in which L-HES was associated with dense infiltration of IgG4-positive plasma cells in the lymph nodes [12]. In this study, we report two cases of clonal rearrangements of TCR genes presenting with HES, elevated circulating IgG4 levels, and kidney injury, providing an opportunity to investigate further the pathological mechanisms underlying the coexistence of elevated IgG4 concentrations and HES.

## 2 Case report

A 60-year-old male was admitted to the nephrology department on January 7, 2017, presenting with edema and an elevated serum creatinine (sCr) level for the past month. He had no history of allergies, exposure to toxins, or the use of specific medications. Physical examination revealed a heart rate of 106 bpm, a respiratory rate of 18 breaths/min, blood pressure of 180/120 mmHg, and a body temperature of 37.8°C. Palpable lymph node enlargement was noted in the left axilla and right groin, though no tenderness was present. Numbness and hypoalgesia were observed in both hands and the lower extremities below the knees. Routine hematological tests showed a white blood cell (WBC) count of 21.7 × 10^9^/L (normal range: 3.5–9.5 × 10^9^/L); neutrophils at 20.4% (normal range: 40–75%); eosinophils at 68.1% (normal range: 0.4–8%); eosinophil count of 14.75 × 10^9^/L (normal range: 0.02–0.52 × 10^9^/L); hemoglobin level of 78 g/L (normal range: 115–150 g/L); and CRP of 6 mg/L. Routine urinary analysis revealed red blood cells (++/HP) and protein (++), with a urinary protein level of 1625.40 mg. Blood biochemistry results were as follows: albumin 21.6 g/L (normal range: 40–55 g/L), creatinine (sCr) 362 μmol/L (normal range: 40–83 μmol/L), uric acid 460 μmol/L (normal range: 140–340 μmol/L), and lactate dehydrogenase 264 U/L. Immunological blood tests are shown in Table 1. Blood and urine amylase levels were within normal limits, and common parasitic-related antibodies tested negative. B19-IgM is negative. The key laboratory results are summarized in Table 1.

**Table 1:** Laboratory characteristics at baseline and post-corticosteroid therapy

Component	Case 1			Case 2			Reference range
	Baseline	Therapy (1 week)	Therapy (2 weeks)	Baseline	Therapy (1 week)	Therapy (4 weeks)	
**Urinalysis**							
Urinary protein	2+	2+	2+	2+	2+	2+	Negative
RBC (n/HPF)	2+	2+	2+	—	—	—	0–3
WBC (n/HPF)	—	—	—	—	3–5	3–5	0–5
UPE (g/24 h)	1.6	2.2	2.1	1.5	1.4	1.4	0–0.2
**Hematology**							
WBC (×10^9^/L)	21.7	9.6	11.8	8.9	9.4	6.9	3.5–9.5
Neutrophils ratio (%)	20.4	84.6	96.8	65.8	81.0	80.5	40–75
Eosinophils (×10^9^/L)	14.75	0.01	0.00	1.51	0.00	0.11	0.02–0.52
Eosinophil ratio (%)	68.1	0.1	0.0	15.9	0.0	1.7	0.4–8
Platelets (×10^9^/L)	266	246	115	125	157	109	125–350
Hemoglobin (g/L)	78	70	79	90	115	109	115–150
CRP (mg/L)	6.0	1.0	1.0	3.8	1.6	1.3	0–8
**Blood chemistry**							
BUN (mmol/L)	16.9	33.3	46.5	41	41.8	35.3	2.6–7.5
Cr (µmol/L)	362	270	571	530	419	391	40–83
Alb (g/L)	21.6	24.5	25.6	31.5	35.2	28.1	40–55
UA (µmol/L)	460	671	516	543	468	416	140–340
**Blood immunology**							
C3 (g/L)	0.89	0.81	0.68	0.63	0.68	0.65	0.70–1.40
C4 (g/L)	0.26	0.16	0.14	0.36	0.29	0.29	0.10–0.40
IgG (g/L)	21.54	18.35	10.81	16.80	10.40	9.57	8.60–17.40
IgG4 (g/L)	8.74	6.78	3.0	2.46	1.80	1.48	0–2.0
IgA (g/L)	1.54	1.50	1.08	4.47	2.88	2.64	1.00–4.20
IgM (g/L)	1.08	1.00	0.97	0.74	0.62	0.57	0.50–2.80
IgE (U/mL)	1414.8	908.3	588.0	266.5	Undetected	252.5	8.6–17.4
RF (U/mL)	68.0	50.0	31.0	Undetected	Undetected	Undetected	0–40
MPO-ANCA	+	+	+	Negative	Negative	Negative	Negative
P-ANCA	+	+	+	Negative	Negative	Negative	Negative
PR3-ANCA	Negative	Negative	Negative	Negative	Negative	Negative	Negative
C-ANCA	Negative	Negative	Negative	Negative	Negative	Negative	Negative
Anti-GBM antibody (RU/mL)	Negative	Negative	Negative	Negative	Negative	Negative	<20
ANA	Negative	Negative	Negative	Negative	Negative	Negative	Negative
Coombs test	Negative	Negative	Negative	Negative	Negative	Negative	Negative
SIFE	Negative	Negative	Negative	Negative	Negative	Negative	Negative
Parasitic-related antibodies	Negative	Negative	Negative	Negative	Negative	Negative	Negative

HPF, High power field; BUN, Blood urea nitrogen; RF, Rheumatoid factor; RBC, Red blood cells; WBC, White blood cells; UPE, Urinary protein excretion; CRP, C-reactive protein; Cr, Creatinine; Alb, Albumin; UA, Uric acid; MPO-ANCA, Myeloperoxidase-anti-neutrophil cytoplasmic antibodies; PR3-ANCA, Proteinase-3-anti-neutrophil cytoplasmic antibodies; p-ANCA, Anti-myeloperoxidase ANCA; c-ANCA, Anti-proteinase-3 ANCA; anti-GBM, Anti-glomerular basement membrane; ANA, Antinuclear antibodies; C3, Complement 3; C4, Complement 4; SIFE, Serum immunofixation electrophoresis.

**Figure 1:**
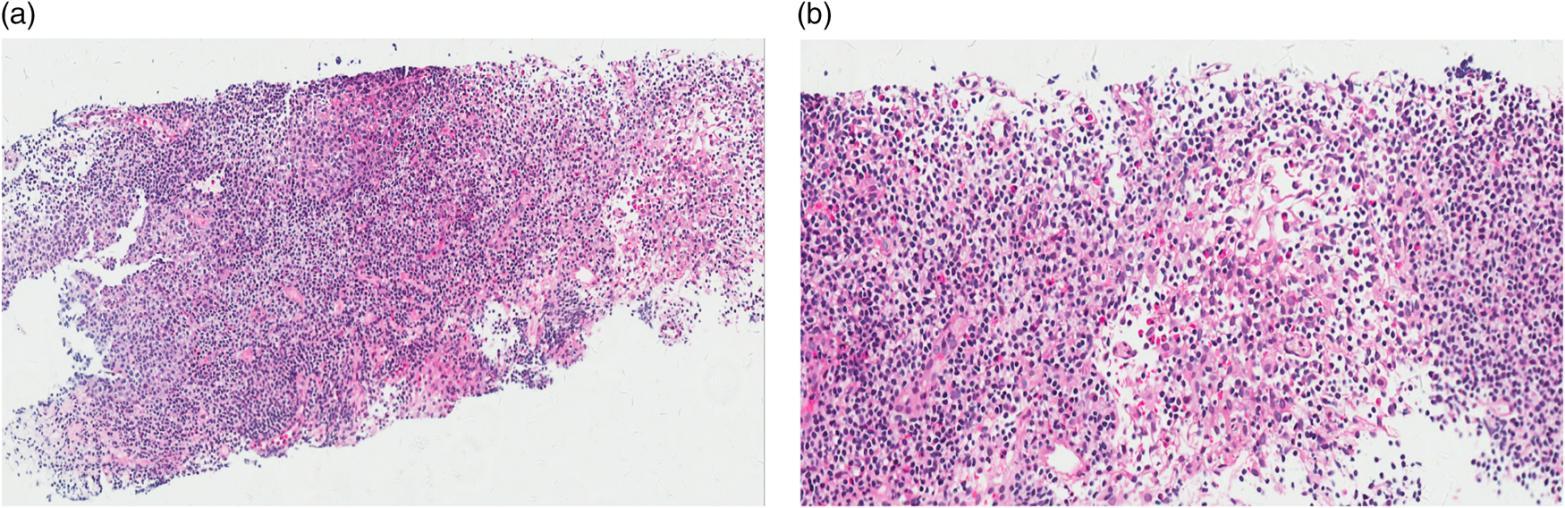
Small lymphoid tissue foci with plasma cells and scattered eosinophilic infiltration. (a) H&E, ×25 and (b) H&E, ×100.

**Figure 2:**
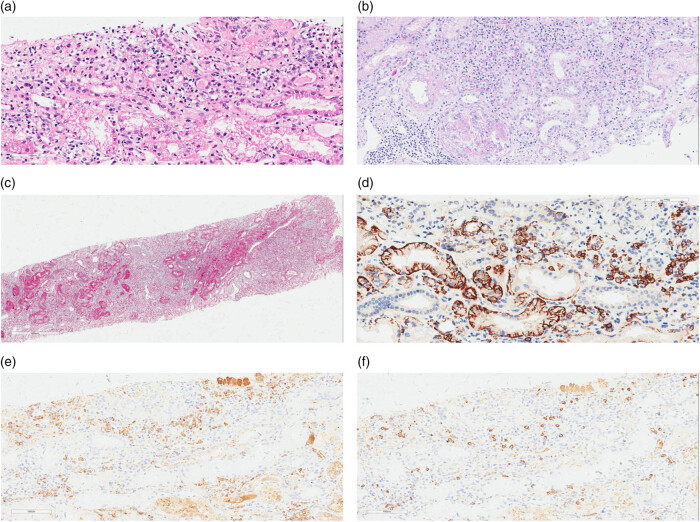
Pathological findings in a renal biopsy specimen. (a) Renal interstitial lesion (H&E, ×100); (b) glomerular abnormalities (PAS, ×200); (c) Renal interstitial lesion (MASSON, ×25); (d) CD38-positive plasma cell infiltrates in the renal interstitium (×100); (e) IgG-positive plasma cell infiltrates in the renal interstitium (×100); and (f) IgG4-positive plasma cell infiltrates in the renal interstitium (×100).

Echocardiography revealed left ventricular enlargement, with a left ventricular ejection fraction of 39%. Abdominal Computed Tomography (CT) showed pancreatic enlargement. Lung CT indicated patchy high-density shadows in the lower lobes of both lungs. Electromyography indicated peripheral nerve injury in the limbs. Routine bone marrow examination revealed an increased proportion of eosinophils (9%), with no abnormalities detected in flow cytometric analysis or hematuria immunofixation electrophoresis. Pathological examination of the bone marrow showed poor hematopoietic tissue proliferation. Lymph node biopsy demonstrated small foci of lymphoid tissue with plasma cells (>10 cells/HPF) and scattered eosinophilic infiltration (Figure 1). Kidney pathology immunofluorescence staining in 4 glomeruli showed the following: IgA (−), IgG (+) in capillary loops, IgM (++) in capillary loops, C3 (+++) in capillary loops, C4 (−), C1q (−), Fg (−). Light microscopy of the glomeruli revealed increased mesangial matrix, segmental parietal epithelial cell hyperplasia, tubular epithelial cell swelling and degeneration, multifocal atrophy, an interstitial plasma cell mass (>10/HP), mononuclear cell infiltration, visible clusters of eosinophilic infiltration, multifocal fibrous tissue proliferation, and an indistinct renal small vessel wall. Immunohistochemical staining showed an IgG4+/IgG+ ratio of >40%. Congo red staining was negative. Electron microscopy revealed vacuolar degeneration of renal tubular epithelial cells (Figure 2a–f). Immunofluorescence did not show any evidence of pauci-immune complexes. The glomeruli predominantly revealed mesangial proliferative lesions with no evidence of vascular fibrotic necrosis. Renal interstitial findings were consistent with IgG4-related interstitial nephritis, accompanied by numerous clusters of eosinophilic infiltration [13].

The key characteristics of this patient included being an elderly male with emaciation, poor general condition, fever, recurrent nasal congestion, and rhinorrhea. He also had multiple lymph node enlargements, severe peripheral nerve damage, nephrotic syndrome with abnormal renal function, severe heart failure, HES, moderate anemia, patchy lung shadows, pancreatic enlargement, significantly elevated IgG4 and IgE levels, slightly elevated rheumatoid factor (RF), low titer positive P-anti-neutrophil cytoplasmic antibodies (ANCA) and myeloperoxidase-ANCA (MPO-ANCA), severe eosinophilic infiltration in the kidney, and IgG4-related interstitial nephritis. Blood and urine immunofixation electrophoresis results were negative, and bone marrow flow cytometry showed no abnormalities. However, monoclonal TCR rearrangement was positive. The patient also presented with fever, anemia, recurrent nasal congestion and rhinorrhea, severe peripheral neuropathy, hyper-eosinophilia, and multiple inflammatory infiltrates in the lungs, without signs of infection. Positive P-ANCA and MPO-ANCA further supported the diagnosis of eosinophilic granulomatosis with polyangiitis (EGPA). However, the renal biopsy did not show evidence of vasculitis, leading to the clinical diagnosis of a mimicked EGPA [14]. The ANCA-positive IgG4-RD patients often show lymph node enlargement, kidney disease, elevated eosinophil counts, and higher levels of serum IgG, IgE, ESR, IgG4 subclass antibodies, and CRP compared to patients with anti-neutrophil cytoplasmic antibody-associated vasculitis (AAV) alone.

This suggests an overlapping syndrome between AAV and IgG4-RD; the etiology and pathological mechanisms remain unclear [15,16]. Interestingly, severe cardiac damage or enlargement is rarely reported in either IgG4-RD or EGPA, making cardiac involvement in this patient atypical. It is hypothesized that eosinophil infiltration in the heart may have contributed to the cardiac lesions. Certain hematological conditions, including clonal hematological diseases, can produce positivity for multiple antibodies and HES. Lymph node biopsy ruled out lymphoma and lymphoproliferative diseases, prompting further molecular analyses to investigate the underlying mechanisms. No abnormal mutations were found in PDGFRA, PDGFRB, FGFR1, JAK2, or FLT3, and clonal rearrangements in B cell analyses (IgH, IgK, IgL) were negative. Similarly, no monoclonal TCR rearrangement was found in the TCR B, but monoclonal TCR gamma and TCR delta (TCRD) rearrangements were detected. This further supports the diagnosis of secondary IgG4-RD and mimicked EGPA. Based on these findings, the patient was diagnosed with probable L-HES, presenting concurrently with IgG4-RD, mimicking EGPA and HES [9,17].

The patient was started on methylprednisolone (40 mg/day) on January 16, 2017. Within 3 days, his circulating eosinophil count returned to normal (from 14.75 to 0.06 × 10^9^/L), and his sCr decreased from 362 to 288 μmol/L. However, after 14 days, his sCr increased to 571 μmol/L, while his IgG concentration decreased to 10.81 g/L (from 21.54 g/L), IgG4 concentration decreased to 3.0 g/L (from 8.74 g/L), and total IgE dropped to 588 U/mL (from 1414.80 U/mL), hemodialysis was initiated.

On February 16, 2017, the patient developed acute respiratory failure and acute heart failure due to a severe pulmonary infection. The patient’s family chose to discontinue further treatment. Despite steroid therapy, the patient showed minimal improvement. Given the rapid progression of the disease and the onset of infections, immunosuppressants or biological agents were not pursued. One week later, the patient succumbed to pulmonary infection and heart failure.

The second case involved a 77-year-old female who had experienced recurrent episodes of systemic rashes and severe pruritus over the past 2 years. She had a 20-year history of hypertension and diabetes. One year before, her serum creatinine level was 280 μmol/L. Her highest peripheral blood eosinophil count was 1.13 × 10^9^/L (normal range: 0.02–0.52 × 10^9^/L), with a hemoglobin level of 90 g/L (normal range: 115–150 g/L) and an IgE concentration of 266.5 IU/mL (normal range: 8.6–17.4 g/L). B-ultrasound imaging showed the left and right kidneys measured 8.6 cm × 4.9 cm and 8.2 cm × 4.5 cm, respectively.

The patient was hospitalized due to progressively rising serum creatinine levels, severe pruritus, and multiple scattered flaky rashes. Her highest serum creatinine level reached 530 μmol/L, with a peak eosinophil count of 1.51 × 10^9^/L, a 24 h urinary protein level of 1.5 g, an IgG4 concentration of 2.458 g/L (normal range: 0–2 g/L), C3 at 0.63 g/L (normal range: 0.7–1.4 g/L), and C4 at 0.36 g/L (normal range: 0.1–0.4 g/L). Tests for common parasitic antibodies were negative. The key laboratory results are summarized in Table 1.

Skin biopsies revealed mild eosinophilic infiltration and eczema-like pathological changes. Lymph node biopsy showed eosinophilic infiltration, IgG4+ plasma cells (>30/HPF), and an IgG4/IgG ratio of less than 40%. Bone marrow analysis showed 5% eosinophils. Serum and urine immunofixation electrophoresis were normal, and blood amylase levels were within the normal range, with no evidence of pancreatic enlargement.

In addition to recurring rashes over several years, the patient displayed signs of acute injury consistent with chronic kidney disease, elevated circulating eosinophil and bone marrow eosinophil counts, enlarged lymph nodes, and increased circulating IgG4 levels. These clinical manifestations were primarily attributed to eosinophil infiltration, with no evidence of organ damage associated with IgG4-RD, ruling out a diagnosis of IgG4-RD. Examination results did not support a parasitic infection. The patient showed hypereosinophilia and eosinophilic infiltration in the lymph nodes and skin, but no symptoms of asthma or sinusitis were present. ANCA tests were negative, excluding a diagnosis of ANCA-associated vasculitis. The primary clinical features were HES and elevated IgG4 levels. Molecular analysis of the bone marrow revealed monoclonal TCRB and TCRD rearrangements, supporting a diagnosis of clonal HES with elevated IgG4 [9,17].

The patient was treated with oral prednisone (20 mg/day), which resolved her rash, pruritus, and normalized eosinophil counts within 3 days. After 7 days, her serum creatinine levels decreased from 530 to 419 μmol/L, prompting a reduction in the prednisone dose to 15 mg/day. Four weeks later, her serum creatinine had further reduced to 391 μmol/L, with an IgG4 concentration of 1.475 g/L and normal eosinophil counts. However, after 3.5 months, her serum creatinine levels rose to 556 μmol/L, the prednisone dose was further reduced to 5 mg/day, and the patient started dialysis. Despite beginning dialysis, her condition remained stable in other aspects, and the prednisone was eventually discontinued. Two years later, the patient passed away due to a lung infection.


**Informed consent:** Informed consent has been obtained from all individuals included in this study.
**Ethical approval:** The research related to human use has been complied with all the relevant national regulations, institutional policies and in accordance with the tenets of the Helsinki Declaration, and has been approved by the Tongde Hospital of Zhejiang Province Ethics Committee.

## 3 Discussion

Both cases described above were diagnosed with HES, featuring monoclonal TCRB and TCRD rearrangements and elevated blood IgG4 concentrations. In the first case, the patient was diagnosed with HES in conjunction with concurrent IgG4-RD, mimicked EGPA and T-cell clonality. However, due to limitations in kidney pathology, it remains unclear whether IgG4-related interstitial nephritis can be definitively confirmed in the second case.

The primary diagnostic test for L-HES involves detecting an aberrant T-cell population through flow cytometry. The most commonly observed T-cell immunophenotype is CD3− CD4+, although other phenotypes, such as CD3+ CD4− CD8− TCRαβ and CD4+ CD7−, have also been reported. These phenotypes can lead to increased IgE production and overproduction of IL-5. In most patients, the size of the abnormal T-cell clone is small and fluctuates with disease activity, making it difficult to detect or confidently validate in routine clinical settings. As a result, further analyses are needed to investigate L-HES in such cases. The most reliable method for detecting this lymphocyte subset involves using an adapted panel of antibodies for flow cytometry, focusing on acquiring numerous events from CD4+ cells. This approach requires specialized expertise from a cytometrist to design the panel and analyze the results. If the small CD3− CD4+ subset is missed using this method or if this approach is not feasible, other tests may be employed to assess the likelihood of L-HES. Standardized tests include serum thymus and activation regulated chemokine measurement by ELISA, analysis of TCR gene rearrangement patterns, and serum IgM measurement [9,18]. Quantification of intracellular cytokines via flow cytometry is not yet standardized and is typically limited to research settings.

Flow cytometry identified no abnormal T-cell immunophenotype in the two cases discussed. Furthermore, serum and urine immunofixation electrophoresis results were normal. Due to experimental limitations at the time, cytokines and abnormal lymphocytes were not further confirmed, representing a limitation in these cases. A previous study reported that only 3 patients out of 18 with evidence of T-cell clonality showed phenotypically abnormal T-cell subsets. Both cases demonstrated clonal TCR gene rearrangements, hypereosinophilia, elevated blood IgG4 levels, and multi-organ involvement. Based on this, we concluded that the clonal TCR gene rearrangements in T-cell populations are relevant to the elevated blood IgG4 levels and disease progression in these patients. Thus, L-HES should be considered in both cases [17,19].

Clonal TCR gene rearrangements can lead to abnormally elevated eosinopoietin levels, potentially mimicking the production of multiple autoantibodies. In patients with autoimmune hemolytic anemia or Evans syndrome, clonal immunoglobulin/T cell receptor gene rearrangements are associated with more severe hemolysis and anemia, requiring longer treatment to achieve remission [20]. However, clonal Ig/TCR gene rearrangements may also be observed in patients with other autoimmune diseases, such as Sjogren’s syndrome. Clonal immunoglobulin heavy chain gene rearrangements have been detected in some patients with systemic lupus erythematosus. Currently, there is no available literature linking TCR rearrangements with ANCA. However, recent case reports and series have described the co-occurrence of IgG4-RD and ANCA-associated vasculitis. IgG4-RD is primarily associated with EGPA and granulomatosis with polyangiitis, although the underlying pathological mechanism remains unclear [20,21].

Hypereosinophilia is commonly observed in IgG4-RD and EGPA, affecting approximately 30% of patients. In EGPA, MPO-ANCA is the predominant ANCA specificity. Pathologically, IgG4-RD is characterized by dense lymphoplasmacytic infiltration, a high proportion of IgG4+ plasma cells, and the predominant production of Th2 cytokines such as IL-4, IL-5, and IL-13. There is also an increase in IL-10 and TGF-β hyper-producing CD4+ CD25+ Foxp3+ T regulatory cells (Tregs). In the pathogenesis of IgG4-RD, eosinophils release various cytokines, chemokines, and growth factors. These eosinophil-derived cytotoxic inflammatory mediators recruit and activate M2 macrophages and fibroblasts, leading to fibroinflammatory infiltration through TGF-β1, PDGF, and IL-13 molecules. Eosinophil dysregulation plays a central role in many eosinophil-associated diseases. Moreover, necrotizing pauci-immune glomerulonephritis in EGPA is marked by significant eosinophil-rich interstitial infiltrates. Eosinophil activation and degranulation contribute to inflammation, which causes structural and functional alterations in tissues. Both eosinophils and IL-5 are critical to the pathogenesis of EGPA, which shows a type 2 inflammatory profile associated with T-helper type 2 (Th2) and T-regulatory cell activity, including increased expression of IL-5, IL-4, and CC motif chemokine receptor 4 [22,23].

IgG4 does not form immune complexes and is inefficient at activating complement. It can also inhibit the proinflammatory functions of Th2 cells. Both IgG4 and IgE are typically produced in response to chronic antigen exposure. The production of IgE and IgG4 is primarily driven by Th2 cytokines such as IL-4 and IL-13. Moreover, IL-10 promotes IgG4 production while inhibiting IgE. Regulatory T cells secrete IL-10, which further stimulates IgG4 production, and a significant proportion of IL-10-producing regulatory B cells also secrete IgG4 [1,2].

In these two cases, the pathological mechanisms involving HES, IgG4-RD, mimicked EGPA, and elevated blood IgG4 levels were interconnected due to clonal TCR rearrangement. Hypereosinophilia played a central role in the pathological process [21,24]. Currently, there is no definitive diagnostic criterion for L-HES, and detecting an aberrant T-cell population via flow cytometry has strict technical requirements regarding cell quantity and expertise. It is possible that similar to the classification criteria for IgG4-RD, a classification system for L-HES could be developed by combining clinical manifestations and laboratory tests with diagnosis based on a scoring system. Differentiating L-HES from IgG4-RD remains a challenge, as elevated circulating eosinophils may either be a manifestation of IgG4-RD or, in some cases, an underlying cause.

Oral corticosteroids are the first-line treatment for L-HES. However, relapses are common when these medications are tapered, often necessitating the use of immunosuppressive therapies. There is no universally accepted treatment for steroid-refractory L-HES. Therapeutic approaches are chosen based on their ability to alleviate symptoms or target the signaling pathways of aberrant T cell clones. Interferon-alpha (IFNα) [19], IL-5 blockade, and various immunosuppressive drugs have been used in a limited number of patients with varying degrees of efficacy [25]. Among these, IL-5 blockade and IFNα are most promising. In the present case, we decided to initiate appropriate corticosteroid treatment for the patient. The first patient, however, did not adhere to the corticosteroid regimen and died from multiple organ failure shortly after developing an infection. The second patient responded to the corticosteroids to some extent but remained dependent on low-dose steroids, eventually passing away from a lung infection after 3 years.

## 4 Conclusion

In conclusion, we present two rare cases of HES associated with T-cell clonality and elevated blood IgG4 levels. The first case was particularly complex, involving T-cell clonality complicated by HES, IgG4-RD, and simulated EGPA. Given the intricate etiology and pathological mechanisms, further pathological and genetic investigations may be required. Developing classification criteria for L-HES could aid in accurate and timely diagnosis and treatment, ultimately improving patient outcomes.
